# Larval competition between the invasive Anopheles stephensi and African native mosquitoes

**DOI:** 10.21203/rs.3.rs-9807896/v1

**Published:** 2026-06-05

**Authors:** Yan Sun, Xiaoming Wang, Kewei Zhang, Guiyun Yan, Guofa Zhou

**Affiliations:** Nanjing Medical University; University of California, Irvine; University of California; University of California, Irvine; University of California, Irvine

**Keywords:** Anopheles stephensi, larval competition, Anopheles arabiensis, Anopheles gambiae sensu stricto, Aedes aegypti, Culex quinquefasciatus

## Abstract

The ecology of the invasive *Anopheles stephensi* in Africa remains poorly understood. Using laboratory strains, we conducted larval competition experiments between *An. stephensi* and African mosquito species under varying conditions of food availability and larval density. When *An. stephensi* was reared alone, high food availability did not influence adult emergence rates; however, emergence time was significantly prolonged at higher larval densities. The low food conditions and increased larval density led to both a significantly reduced emergence rate and a longer development time. When *An. stephensi* was co-reared with *Anopheles arabiensis*, its emergence rate was significantly reduced (31% reduction) compared with when reared alone; however, the decline in emergence rate was more pronounced in *An. arabiensis* (64% reduction). Similarly, co-rearing of *An. stephensi* and *Anopheles gambiae* sensu stricto only significantly reduced the emergence rate of adult *An. gambiae* (46% reduction). Across these experiments, changes in emergence time were generally not significant for either species. Larval competition between *An. stephensi* and *Aedes aegypti* had minimal effects on the emergence rate and development time of *Ae. aegypti*, however, it substantially reduced the adult emergence rate of *An. stephensi* under all experimental conditions (88% reduction, range 75–100%). Co-rearing *An. stephensi* with *Culex quinquefasciatus* had no significant effects on either emergence rates or development times for either species. The results suggest that *An. stephensi* may outcompete African malaria vectors but is strongly outcompeted by *Ae. aegypti*.

## Introduction

Invasive mosquito species can reshape the local mosquito ecological environment by competing with native species for resources such as food and space, thereby exerting significant impacts on the transmission and control of local mosquito-borne diseases (Yan et al., 2024). *Anopheles stephensi* (Diptera: Culicidae) Liston, 1901 is a major malaria vector originally from South and Southeast Asia (Sinclair et al., 2011). It spread to the Middle East in the 1980s and reached Arabian Peninsula in the late 2000s (Ishtiaq et al., 2021; Sinka et al., 2011). The species was first detected in Djibouti in the Horn of Africa in 2012 (Faulde et al., 2014), and it has since spread to all countries in the region as well as several countries in West Africa (WHO, 2022; Zhou et al., 2024). Unlike Africa’s indigenous malaria vectors, which are primarily adapted to rural environment, *An. stephensi* thrives in urban settings (Balkew et al., 2021, Zhou et al., 2024). The invasion of *An. stephensi* to Africa has been linked to malaria outbreaks in Djibouti and Ethiopia (de Santi *et al*., 2021; Emiru et al., 2023). Modeling studies estimate a ~ 50% increase in malaria prevalence in Ethiopia and an additional 126 million people at risk for malaria in Africa (Hamlet et al., 2022; Sinka et al., 2020). This escalating threat has prompted the World Health Organization (WHO) to call for urgent action to contain its spread in Africa (WHO, 2022). However, major knowledge gaps in the ecology and behaviors of *An. stephensi* continue to hinder the development of effective mosquito surveillance and control strategies, impeding efforts to curb its expansion and design cost-effective malaria interventions (Hemming-Schroeder and Ahmed, 2023; Ishtiaq et al., 2021, Zhou et al., 2024).

*Anopheles stephensi* exhibits distinct ecological patterns between its native range in Asia and its invaded range in Africa. In its native region of Asia, *An. stephensi* is commonly found in rural as well as urban areas, and breeds in both artificial and natural habitats (Amani et al., 2014; Amini et al., 2020; Edalat et al., 2020; Sinka et al., 2011). For example, Amani *et al*. only found *An. stephensi* in natural habitats and none in artificial habitats in Aligudarz County, western Iran (Amani et al., 2014), while Thomas *et al*. found *An. stephensi* larval primarily from artificial habitats such as water tanks/containers and wells in Chennai, a major city in southern India (Thomas et al., 2016). In addition, *An. stephensi* coexists with other Asian native malaria vectors in a large proportion of larval habitats (Amani et al., 2014; Amini et al., 2020; Sinka et al., 2011; Thomas et al., 2016; Zhou et al., 2024). For example, in southern Iran along the Persian Gulf coast, Vatandoost *et al*. found that *An. stephensi* larvae shared habitats with several local anopheline species such as *Anopheles fluviatilis*, *Anopheles culicifacies*, *Anopheles superpictus*, *Anopheles turkhudi* and *Anopheles mongolensis* (Vatandoost et al., 2006). In Africa, current studies indicate that the primary breeding habitats for *An. stephensi* have been found as man-made artificial habitats located in urban areas, and *An. stephensi* larvae rarely share habitats with native African malaria vectors (Ahmed et al., 2021; Balkew et al., 2020, 2021; PMI, 2020, 2023). The reasons behind the rare coexistence of *An. stephensi* larvae with native vector species larvae in Africa remain unclear.

Multiple mechanisms mediate species coexistence or segregation and invasion performance in a community: 1) gravid *An. stephensi* females may avoid laying eggs in habitats already occupied by native malaria vector species to avoid inter-specific larvae competitions; 2) competitive exclusion of *An. stephensi* by native African mosquitoes or vice versa; and/or, 3) other environmental and ecological factors, i.e., potential niche divergence or segregation (Hui et al., 2023). As an invasive species in Africa, *An. stephensi* must compete with native African *Anopheles* and other mosquitoes, overcoming biotic resistance from local mosquito community to establish and sustains stable populations (Evans et al., 2021; Ezeakacha and Yee, 2019; Haq et al., 2019; Marini et al., 2017; Noden et al., 2016; Talaga et al., 2018). The impact of local vector community structure on the habitat selection and larval survival of *An. stephensi* in African environments remains unknown (Bonhomme et al., 2021; Capdevila et al., 2021; Juliano, 2009).

Despite the importance of competition, larval interactions between *An. stephensi* and native African mosquito species remain poorly understood. Evidence from Asia suggests that *An. stephensi* is a weaker competitor than *Ae. aegypti* (Evans et al., 2021; Haq et al., 2019), but it is unclear whether this pattern holds in African mosquito communities. Native vectors such as *Anopheles arabiensis* and *Anopheles gambiae* sensu stricto frequently exploit similar artificial container habitats, indicating a high potential for competition with *An. stephensi* (Degefa et al., 2025). Although *Culex quinquefasciatus* is widespread in urban Africa, its competitive relationship with *An. stephensi* has not been characterized. Nonetheless, the mechanisms underlying the invasion success and population persistence of *An. stephensi* in Africa remain largely unexplored.

This study aims to determine whether larval competition between *An. stephensi* and native African mosquito species can lead to competitive exclusion, thereby enabling *An. stephensi* to sustain its populations in Africa, and thus explains the apparent segregation of larval habitats among these species observed in the field. By comparing interspecific and intraspecific interaction strengths, we evaluated the effects of food availability and rearing density on larval competitive interactions between *An. stephensi* and major mosquito species in Africa, including *An. arabiensis*, *An. gambiae* s.s., *Ae. aegypti*, and *Cx. quinquefasciatus*, under laboratory conditions. We hypothesize that *An. stephensi* can outcompete African malaria vectors, facilitating its establishment and persistence in African environments. Addressing these gaps is essential for predicting the future spread of this species, assessing its potential impact on malaria transmission, and informing the development of effective control strategies.

## Materials and Methods

### Mosquito strains and experimental conditions

All experimental mosquito populations were obtained from laboratory strains maintained by BEI Resources (Manassas, Virginia 20110, USA), including *An. stephensi*, *An. arabiensis*, *An. gambiae* s.s., *Ae. aegypti*, and *Cx. quinquefasciatus*. All experiments were conducted at controlled laboratory conditions, with a temperature of 26 ± 1°C, humidity of 70 ± 10%, and light : dark condition of 12:12 h. Larvae were reared in 500ml cups with 100ml of distilled water regardless of larval density; additional water was supplied to keep a stable water volume. Larvae were reared with two food supply types, i.e., 1.5mg/cup (low food condition) or 3mg/cup (high food condition) every other day.

### Impact of larval density and food supply on An. stephensi larval development

One-day-old larvae were reared at densities of 10, 20, 40, and 60 per 100ml water with both food supplies. For each treatment, three replicates were conducted. Larvae, pupae, and adults were counted every other day until all larvae emerged or died. The emerged adults were separately counted for males and females. The wing size, a representation of body size, of all emerged adults was measured.

### Larval competitions between An. stephensi and other mosquitoes

Larval competition experiments were conducted between *An. stephensi* and other mosquito species, including *An. arabiensis*, *An. gambiae*, *Ae. aegypti*, and *Cx. quinquefasciatus*. Larvae competitions were conducted at densities of 20 and 60 per 100ml water with both food supplies. The selection of 20 and 60 larvae per 100ml of water was based on the results of *An. stephensi* larval density experiments, i.e., the two densities showed significantly different adult emergence rates and emergence times. All species, including *An. stephensi*, were reared at two conditions, i.e., either reared alone or mixed of two species with equal numbers at 20/60 larvae/100 ml water. All species started with one-day-old larvae. Larvae, pupae, and adults (separately for males and females) were counted every other day until all larvae emerged or died, with a maximum observation period of 25 days. Larvae of *Anopheles* species were not identified as species, and the emerged adults were morphologically identified as species. All experiments were conducted with three replicates.

### Data analysis

The primary outcomes were adult emergence rate and emergence time for all experiments. The wing size was measured for *An. stephensi* density-dependent survival experiments. For *An. stephensi*, emergence rate (after arcsine transformation), emergence time (separately for males and females), and wing size (separately for males and females) were compared using analysis of variance (ANOVA) for the same food supply but different larval densities, and pairwise comparison was performed using the post hoc Tukey-Kramer HSD test. A t-test assuming unequal variance was used to examine the differences for the same three parameters between different food supplies at the same larval density. The generalized linear model was used to examine the impact of food supply and larval density on adult emergence rate, emergence time, and wing size.

For the competition experiments, emergence rate (after arcsine transformation) and emergence time (analyzed separately for males and females) were compared across all species between single-species rearing and mixed-species rearing conditions. The impact of interspecific competition between *An. stephensi* and African mosquito species was assessed by quantifying the relative change (reduction or increase) in emergence rate when species were reared in mixed-species conditions compared with single-species conditions. To determine whether any species was more strongly affected by competition, changes in emergence rates were compared between the two competing species using a t-test assuming unequal variances. Since three of the four competition experiments had some zero adult emergences, which resulted in only a few data points for emergence time; therefore, generalized linear model analysis was not performed for competition outcomes.

## Results

### Impact of larval density and food supply on An. stephensi larval development

#### Adult emergence rates.

**Figure 1:**
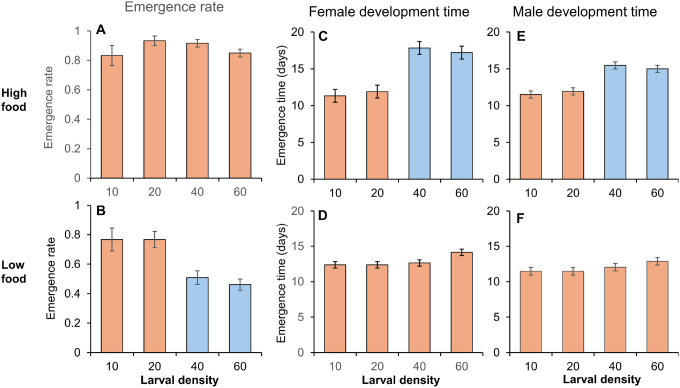
*Anopheles stephensi*adult emergence rate and emergence time at different larval density settings. Different colors of bars within the same figure represent significantly different from each other at a level of 0.05.

At high food supply, average adult emergence rates ranged from 0.83 to 0.93 for different larval densities, and there were no significant differences in adult emergence rates among different larval densities (ANOVA post hoc Tukey-Kramer test, P > 0.05, Fig. 1A). In contrast, at low food supply, larvae reared at higher densities of 40/60 larvae/100 ml water significantly reduced adult emergence rates (average of 48.4%) compared to 76.6% at lower larvae densities of 10/20 larvae/100 ml water (ANOVA post hoc Tukey-Kramer test, P < 0.05, Fig. 1B).

#### Adult emergence times.

Conversely, low food supply did not affect adult emergence times regardless of larval rearing densities (ANOVA post hoc Tukey-Kramer test, P > 0.05, Figs. 1D & 1F). However, at high food supply, higher densities of 40/60 larvae/100 ml water significantly increased adult emergence times by about 6 days for females and 3.5 days for males compared to larvae reared at 10/20 larvae/100 ml water (ANOVA post hoc Tukey-Kramer test, P < 0.05, Figs. 1C & 1E).

#### Adult wing size.

Both food supplies and larval densities affected adult wing size of both males and females (supplement Figure S1). In general, the higher the density, the shorter the wing size regardless of sex and food supplies (ANOVA post hoc Tukey-Kramer test, P < 0.05, supplement Figure S1). On the other hand, high food supplies resulted in significantly larger adult wing size except for larval density of 60 larvae/100 ml water (t-test assuming non-equal variance, P = 0.05, supplement Table S1).

#### Multivariate analysis.

The results from multivariate analysis showed that both food supplies and larval densities and their interactions significantly affected adult emergence rates and emergence times (supplement Tables 2 & 3). In general, density had a negative effect, and food supply had a positive effect on adult emergence rates, and the inverse is true for the adult emergence times (supplement Tables 2 & 3).

### Laval competition between An. stephensi and An. arabiensis

**Figure 2:**
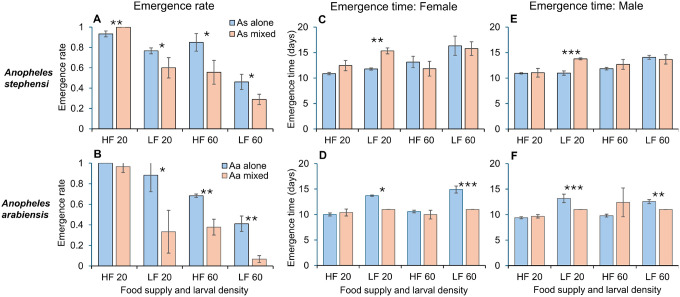
Adult emergence rate and emergence time of *Anopheles stephensi* (top panel) and *Anopheles arabiensis* (bottom panel) when each species was reared alone (light blue bars) and when they were reared as mixed (orange color bars) at different larval density and food supply settings. HF and LF represent high food and low food supplies, 20 and 60 followed by HF/LF represent larval densities of 20 and 60. *, **, and *** represent significantly different between the two rearing conditions at levels of 0.05, 0.01 and 0.001.

The adult emergence rate of both species was not affected by high food supply at larval density of 20 larvae/100 ml water; otherwise, the emergence rates of both species were significantly reduced when the two species were reared together (Figs. 2A & 2B). However, the reduction in emergence rate was more pronounced for *An. arabiensis* (63.6%) compared to *An. stephensi* (31.2%) (t = 2.63, d.f. = 3, P = 0.039).

When the two species were reared together, there were some significant increases in emergence time (for both females and males) for *An. stephensi* while there were some significant decreases in emergence time (for both females and males) for *An. arabiensis*, but in most cases, competition had minor effects on adult emergence times for both species (t-tests, P = 0.05) (Fig. 2C–F).

### Laval competition between An. stephensi and An. gambiae s.s.

**Figure 3:**
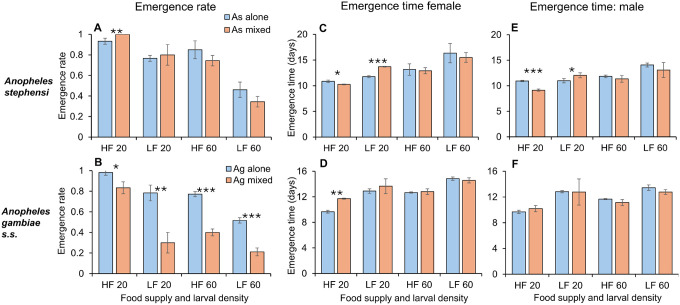
Adult emergence rate and emergence time of *Anopheles stephensi* (top panel) and *Anopheles gambiae* s.s. (bottom panel) when each species was reared alone (light blue bars) and when they were reared as mixed (orange color bars) at different larval density and food supply settings. HF and LF represent high food and low food supplies, 20 and 60 followed by HF/LF represent larval densities of 20 and 60. *, **, and *** represent significantly different between the two rearing conditions at levels of 0.05, 0.01 and 0.001.

The effects of competition on adult emergence rates and emergence times between *An. stephensi* and *An. gambiae* were primarily on *An. gambiae* (Fig. 3). In most cases, the competition slightly affects the emergence rate of *An. stephensi* (average reduction 6.6%), while the emergence rate of *An. gambiae* was significantly reduced for all experiments (average reduction 46.1%) (t = 3.01, d.f. = 5, P = 0.015, Figs. 3A & 3B).

Meanwhile, larval competition resulted in a minor increase in adult emergence times for both species under all conditions (t-tests, P = 0.05) (Fig. 3C–F).

### Laval competition between An. stephensi and Ae. aegypti

**Figure 4:**
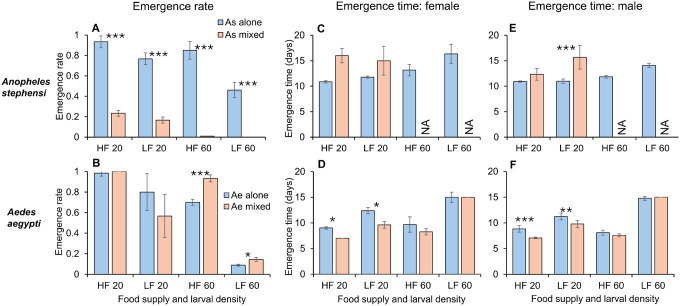
Adult emergence rate and emergence time of *Anopheles stephensi* (top panel) and *Aedes aegypti* (bottom panel) when each species was reared alone (light blue bars) and when they were reared as mixed (orange color bars) at different larval density and food supply settings. NA means not applicable. HF and LF represent high food and low food supplies, 20 and 60 followed by HF/LF represent larval densities of 20 and 60. *, **, and *** represent significantly different between the two rearing conditions at levels of 0.05, 0.01 and 0.001.

Remarkably, larval competition between *An. stephensi* and *Ae. aegypti* severely reduced the emergence rates of *An. stephensi* (average reduction 88.0%), but it had little effect on that of *Ae. aegypti* (17.1% increase) (t = 5.04, d.f. = 4, P = 0.0036) (Figs. 4A & 4B). When *An. stephensi* and *Ae. aegypti* were reared together, only a single *An. stephensi* male emerged, and this occurred in the high-food, high-density treatment; no *An. stephensi* adults emerged under low-food conditions. At low larval density, *An. stephensi* adult emergence remained very low (< 10%) across all food levels. Since most of the competition experiments resulted in no emergence of *An. stephensi*, it is difficult to compare emergence times for *An. stephensi* between experiments when it was reared alone and when it was reared together with *Ae. aegypti*. Nonetheless, adult emergence times of *Ae. aegypti* significantly decreased in some cases, while there was no significant effect in other cases (t-tests, P = 0.05) (Figs. 4C–F).

### Laval competition between An. stephensi and Cx. quinquefasciatus

**Figure 5:**
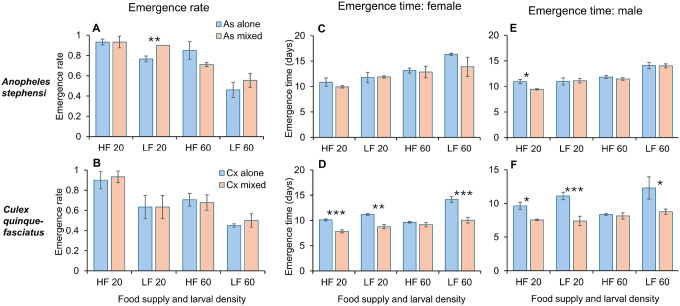
Adult emergence rate and emergence time of *Anopheles stephensi* (top panel) and *Culex quinquefasciatus* (bottom panel) when each species was reared alone (light blue bars) and when they were reared as mixed (orange color bars) at different larval density and food supply settings. HF and LF represent high food and low food supplies, 20 and 60 followed by HF/LF represent larval densities of 20 and 60. *, **, and *** represent significantly different between the two rearing conditions at levels of 0.05, 0.01 and 0.001.

Interestingly, larval competition between *An. stephensi* and *Cx. quinquefasciatus* had very little impact on the adult emergence rates of both species (t-test, P = 0.05, Figs. 5A&B). Larval competition had no significant effect on the adult emergence time of *An. stephensi*, however, surprisingly mixed rearing resulted in significantly shorter emergence time for *Cx. quinquefasciatus* in half of the experiments (t-tests, P = 0.05) (Figs. 5C–F).

## Discussion

*Anopheles stephensi* has successfully established its population in the Horn of Africa since its first detection in Djibouti in 2012 (Balkew et al., 2021; Faulde et al., 2014). For an invasive vector to persist in a novel range, *An. stephensi* must find its suitable niche to sustain its population and withstand biotic interactions with native species. In both urban and rural Africa, there are many indigenous mosquito species, for example, *An. gambiae* s.s., *An. arabiensis*, *Cx. quinquefasciatus* in rural areas, and *An. arabiensis*, *Ae. aegypti*, and *Cx. quinquefasciatus* in urban areas (Carter et al., 2022; Eba et al., 2021; Mwingira et al., 2021; Takken et al., 2024; Tenywa et al., 2022). Beyond differences in local environments between Asia and Africa, *An. stephensi* must either establish a suitable ecological niche or outcompete native African mosquito species to sustain its population in Africa. In other words, success in interspecific competition is one of the key strategies for its survival in Africa. So far, the competition between larvae of *An. stephensi* and African mosquitoes have not been studied (Zhou et al., 2024). Here, based on laboratory experiments, we found that *An. stephensi* significantly outcompetes the African malaria vectors *An. arabiensis* and *An. gambiae* s.s.. However, it performed poorly against the urban container–inhabiting mosquito *Ae. aegypti* and shows little to no competitive interaction with the widely distributed *Cx. quinquefasciatus*. These findings advance our understanding of invasion ecology, though they should be interpreted with caution, as validation using field populations is needed.

The results from this study suggested that *An. stephensi* may survive well in rural Africa because it outcompetes the major rural malaria vectors, including *An. gambiae* s.s. and *An. arabiensis*. In urban Africa, *An. stephensi* has thus far been found primarily in large water storage tanks (used for drinking and other purposes) and in water-filled manmade ponds (either concrete or plastic) at construction or brickmaking sites (Ashine et al., 2024; Balkew et al., 2021). In contrast, *Ae. aegypti* is mainly found in small water containers and is less commonly observed in large water tanks in places other than Ethiopia (Kahamba et al., 2020; Musunzaji et al., 2023). In Ethiopia, *Ae. aegypti* has also been found in large water-holding containers such as cemented cisterns (Seid et al., 2024; Yared et al., 2024). The results of larval competition between *Ae. aegypti* and *An. stephensi* in this study are consistent with previous findings demonstrating asymmetric competition, with *Ae. aegypti* as the dominant competitor (Evans et al., 2021). Our experiments yielded similar patterns. Likewise, Haq et al. reported a competitive advantage of *Ae. aegypti* over *An. stephensi*, with outcomes that are dependent on food availability and larval density, in line with our observations (Haq et al., 2019). However, our three-year field surveys indicate that cohabitation of *Ae. aegypti* and *Anopheles* larvae is rare in eastern Ethiopia (Zhou, unpublished data), suggests limited niche overlap in larval habitats between the *Ae. aegypti* and *An. stephensi*. Taken together, these findings suggest that ecologically, *An. stephensi* has a strong potential to establish and sustain its populations in both urban and rural Africa. However, the results should be interpreted with caution and require further validation with field mosquito populations under natural conditions. To our knowledge, this is the first experimental study demonstrating that *An. stephensi* can outcompete native African malaria vectors, providing an ecological basis for its potential spread across the continent.

These results have important implications for malaria vector ecology, as interspecific competition can lead to the displacement or replacement of local species. The interaction between *Aedes albopictus* and *Ae. aegypti* provides a well-documented example of competitive exclusion. In southern Florida, USA, *Ae. aegypti* was the predominant *Aedes* species in the 1980s, prior to the invasion of *Ae. albopictus* in 1988; by 1993, *Ae. aegypti* had largely disappeared, although it has shown signs of localized recovery since 2019 (Brennan et al., 2021; Parker *et al*., 2019). Similarly, in Hainan Province and the Leizhou Peninsula of Guangdong Province, China, *Ae. aegypti* was the dominant *Aedes* species in the 1990s but was gradually displaced by *Ae. albopictus*, and recent surveys report that *Ae. aegypti* is now rarely detected on Hainan Island and in Leizhou peninsula (Li et al., 2021; Zhao et al., 2023). In Dire Dawa, eastern Ethiopia, *An. arabiensis* was historically the dominant urban malaria vector (Degefa et al., 2025). However, recent investigations have shown that *An. stephensi* is now significantly more abundant than *An. arabiensis* in both urban and rural areas (Degefa et al., 2025; Merga et al., 2024). In this region, *An. arabiensis* is also frequently found in man-made habitats (Degefa et al., 2025) (Zhou unpublished data), which are preferred breeding sites for *An. stephensi*. In fact, several studies reported that *An. stephensi* is the predominant *Anopheles* species in many other sites across Ethiopia (Ashine et al., 2024; Degefa et al., 2025; Zhou et al., 2024). Whether *An. stephensi* will displace *An. arabiensis* in Africa, and the relative contributions of each species to malaria transmission in these areas, remain important questions for future research. However, the interspecific competition results from this study suggest that *An. stephensi* has the potential to replace native African *Anopheles* species in invaded regions.

The main limitations of this study are the use of laboratory mosquito strains under controlled laboratory conditions. The findings would be more robust if similar experiments were conducted using field-collected mosquitoes from Africa. The use of distilled water and artificial larval food minimizes confounding factors such as variation in the physicochemical properties of natural breeding waters; and most laboratory competition studies are conducted under such standardized conditions (Evans et al., 2021; Giatropoulos et al., 2022). Conversely, using water from different natural habitats could provide a clearer understanding of how habitat type influences larval development, survival, and competitive outcomes (Bennett et al., 2021; Reiskind et al., 2012). Such an approach would also enable investigation of oviposition preferences, thereby improving our understanding of mosquito reproductive behavior in addition to interspecific competition (Asmare et al., 2017; Eneh et al., 2019; Musunzaji et al., 2023). Collectively, these unresolved questions, along with the potential for intraspecific larval cannibalism in *An. stephensi*, highlight the need for further studies using field-collected mosquitoes under natural field conditions.

In conclusion, our experiments demonstrate that *An. stephensi* outcompetes native African malaria vectors but is strongly outcompeted by *Ae. aegypti*. These findings provide an ecological basis for the invasion and spread potential of *An. stephensi* in Africa. Its competitive advantage over native African malaria vectors may facilitate its establishment and expansion, particularly in rural areas of Africa. Conversely, its competitive disadvantage relative to *Ae. aegypti* may drive habitat partitioning and selection of alternative breeding sites and may limit co-transmission of malaria and arboviruses. These findings provide a critical ecological and epidemiological foundation for understanding the invasion of *An. stephensi* and for developing sustainable vector control strategies in Africa.

## Supplementary Files

This is a list of supplementary files associated with this preprint. Click to download.

- Supplementlegends.docx
- FigureS1wingsize.pptx

## Data Availability

The data underlying this publication is available freely upon request, complying with the data access policies of participated institutions.

## Key Message

The invasion and rapid spread of, a major malaria vector originally from South and Southeast Asia, in Africa has been linked to malaria outbreaks in Djibouti and Ethiopia in east Africa. Unlike Africa’s indigenous malaria vectors, which are primarily adapted to rural environment, thrives in urban settings. This escalating threat has prompted the World Health Organization (WHO) to call for urgent action to contain its spread in Africa. However, major knowledge gaps in the ecology and behaviors of continue to hinder the development of effective mosquito surveillance and control strategies, and impede efforts to curb its expansion in Africa. We conducted larval competition experiments between and African mosquito species and try to understand the mechanisms of population establishment and sustainability in Africa. We found that, under the laboratory conditions, larval could outcompete against Africa native malaria vectors such as and sensu stricto, however, it was outcompeted by larvae, and there is no larvae competition between and the most common mosquito species found in both rural and urban east Africa. These results indicated the competitive advantages (lack of competitive pressure on habitat selection) of against African native malaria vectors, which partly explains its rapid distribution expansion in Africa.

## References

[1] Ahmed A, Pignatelli P, Elaagip A, Abdel Hamid MM, Alrahman OF, Weetman D (2021) Invasive malaria vector *Anopheles stephensi* mosquitoes in Sudan, 2016–2018. Emerg Infect Dis 27:2952–2954. 10.3201/eid2711.210040 34670658 PMC8544976

[2] Amani H, Yaghoobi-Ershadi MR, Kassiri H (2014) The ecology and larval habitats characteristics of anopheline mosquitoes (Diptera: Culicidae) in Aligudarz County (Luristan province, western Iran). Asian Pac J Trop Biomed 4:S233–S241. 10.12980/APJTB.4.2014C186 25183088 PMC4025324

[3] Amini M, Hanafi-Bojd AA, Aghapour AA, Chavshin AR (2020) Larval habitats and species diversity of mosquitoes (Diptera: Culicidae) in West Azerbaijan Province, Northwestern Iran. BMC Ecol 20:60. 10.1186/s12898-020-00328-0 33213441 PMC7677836

[4] Ashine T, Eyasu A, Asmamaw Y, (2024) Spatiotemporal distribution and bionomics of *Anopheles stephensi* in different eco-epidemiological settings in Ethiopia. Parasit Vector 17:166. 10.1186/s13071-024-06243-3 PMC10983662 38556881

[5] Asmare Y, Hill SR, Hopkins RJ, Tekie H, Igenll R (2017) The role of grass volatiles on oviposition site selection by *Anopheles arabiensis* and *Anopheles coluzzii*. Malar J 16:65. 10.1186/s12936-017-1717-z 28173804 PMC5297170

[6] Balkew M, Mumba P, Dengela D, Yohannes G, Getachew D, Yared S, Chibsa S, Murphy M, George K, Lopez K, Janies D, Choi SH, Spear J, Irish SR, Carter TE (2020) Geographical distribution of *Anopheles stephensi* in eastern Ethiopia. Parasit Vector 13:35. 10.1186/s13071-020-3904-y PMC6971998 31959237

[7] Balkew M, Mumba P, Yohannes G, (2021) An update on the distribution, bionomics, and insecticide susceptibility of *Anopheles stephensi* in Ethiopia, 2018–2020. Malar J 20:263. 10.1186/s12936-021-03801-3 34107943 PMC8189708

[8] Bennett KL, McMillan WO, Enríquez V, Barraza E, Díaz M, Baca B, Whiteman A, Cerro Medina J, Ducasa M, Gómez Martínez C, Almanza A, Rovira JR, Loaiza JR (2021) The role of heterogenous environmental conditions in shaping the spatiotemporal distribution of competing *Aede*s mosquitoes in Panama: implications for the landscape of arboviral disease transmission. Biol Invasions 23:1933–1948. 10.1007/s10530-021-02482-y 34776763 PMC8550678

[9] Bonhomme C, Céréghino R, Carrias JF, Compin A, Corbara B, Jassey VEJ, Leflaive J, Farjalla VF, Marino NAC, Rota T, Srivastava DS, Leroy C (2021) *In situ* resistance, not immigration, supports invertebrate community resilience to drought intensification in a Neotropical ecosystem. J Anim Ecol 90:2015–2026. 10.1111/1365-2656.13392 33232512

[10] Brennan SA, Grob IC, Bartz CE, Baker JK, Jiang Y (2021) Displacement of *Aedes albopictus* by *Aedes aegypti* in Gainesville, Florida. J Am Mosq Control Assoc 37:93–97. 10.2987/20-6992.1 34184045

[11] Capdevila P, Stott I, Oliveras Menor I, Stouffer DB, Raimundo RLG, White H, Barbour M, Salguero-Gomez (2021) Reconciling resilience across ecological systems, species and subdisciplines. J Ecol 109:3102–3113. 10.1111/1365-2745.13775

[12] Carter TE, Gebresilassie A, Hansel S, Damodaran L, Montgomery C, Bonnell V, Lopez K, Janies D, Yared S (2022) Analysis of the knockdown resistance locus (kdr) in *Anopheles stephensi*, *An. arabiensis*, and *Culex pipiens* s.l. for insight into the evolution of target-site pyrethroid resistance in Eastern Ethiopia. Am J Trop Med Hyg 106:632–638. 10.4269/ajtmh.20-1357 35008054 PMC8832926

[13] de Santi VP, Khaireh BA, Chiniard T, Pradines B, Taudon N, Larréché S, Mohamed AB, de Laval F, Berger F, Gala F, Mokrane M, Benoit N, Malan L, Abdi AA, Briolant S (2019) Role of *Anopheles stephensi* mosquitoes in malaria outbreak, Djibouti, 2019. Emerg Infect Dis 27:1697–1700. 10.3201/eid2706.204557 PMC8153885 34013869

[14] Degefa T, Zhong D, Lee MC, Merga H, Abiy E, Wang X, Zhou G, Kifle T, Yewhalaw D, Yan G (2025) Bionomics of *Anopheles stephensi* across the urban-rural landscapes of Eastern Ethiopia. Malar J 24:274. 10.1186/s12936-025-05527-y 40849467 PMC12375276

[15] Eba K, Habtewold T, Yewhalaw D, Christophides GK, Duchateau L (2021) *Anopheles arabiensis* hotspots along intermittent rivers drive malaria dynamics in semi-arid areas of Central Ethiopia. Malar J 20:154. 10.1186/s12936-021-03697-z 33731115 PMC7971958

[16] Edalat H, Mahmoudi M, Sedaghat MM, Moosa-Kazemi SH, Kheirandish S (2020) Ecology of malaria vectors in an endemic area, southeast of Iran. J Arthropod Borne Dis 14:325–343. 10.18502/jad.v14i4.5270 33954207 PMC8053069

[17] Emiru T, Getachew D, Murphy M, (2023) Evidence for a role of *Anopheles stephensi* in the spread of drug- and diagnosis-resistant malaria in Africa. Nat Med 29:3203–3211. 10.1038/s41591-023-02641-9 37884028 PMC10719088

[18] Eneh LK, Fillinger U, Borg Karlson AK, Kuttuva Rajarao G, Lindh J (2019) *Anopheles arabiensis* oviposition site selection in response to habitat persistence and associated physicochemical parameters, bacteria and volatile profiles. Med Vet Entomol 33:56–67. 10.1111/mve.12336 30168151 PMC6359949

[19] Evans MV, Drake JM, Jones L, Murdock CC (2021) Assessing temperature-dependent competition between two invasive mosquito species. Ecol Appl 31:e02334. 10.1002/eap.2334 33772946

[20] Ezeakacha NF, Yee DA (2019) The role of temperature in affecting carry-over effects and larval competition in the globally invasive mosquito *Aedes albopictus*. Parasit Vectors 12:123. 10.1186/s13071-019-3391-1 30890161 PMC6423813

[21] Faulde MK, Rueda LM, Khaireh BA (2014) First record of the Asian malaria vector *Anopheles stephensi* and its possible role in the resurgence of malaria in Djibouti, Horn of Africa. Acta Trop 139:39–43. 10.1016/j.actatropica.2014.06.016 25004439

[22] Giatropoulos A, Papachristos D, Michaelakis A, Kapranas A, Emmanouel N (2022) Laboratory study on larval competition between two related mosquito species: *Aedes* (*Stegomyia*) *albopictus* and *Aedes* (*Stegomyia*) *cretinus*. Acta Trop 230:106389. 10.1016/j.actatropica.2022.106389 35276061

[23] Zhou G, Taffese HS, Zhong D, Wang X, Lee MC, Degefa T, Getachew D, Haileselassie W, Hawaria D, Yewhalaw D, Yan G (2024) Resurgence of clinical malaria in Ethiopia and its link to *Anopheles stephensi* invasion. Pathogens 13:748. 10.3390/pathogens13090748 39338938 PMC11435327

[24] Hamlet A, Dengela D, Tongren JE, Tadesse FG, Bousema T, Sinka M, Seyoum A, Irish SR, Armistead JS, Churcher T (2022) The potential impact of *Anopheles stephensi* establishment on the transmission of *Plasmodium falciparum* in Ethiopia and prospective control measures. BMC Med 20:135. 10.1186/s12916-022-02324-1 35440085 PMC9020030

[25] Haq S, Kumar G, Dhiman RC (2019) Interspecific competition between larval stages of *Aedes aegypti* and *Anopheles stephensi*. J Vector Borne Dis 56:303–307. 10.4103/0972-9062.302032 33269729

[26] Hemming-Schroeder E, Ahme A (2023) *Anopheles stephensi* in Africa: vector control opportunities for cobreeding *An. stephensi* and *Aedes* arbovirus vectors. Trend Parasitol 39:86–90. 10.1016/j.pt.2022.11.011 36522231

[27] Hui C, Pyšek P, Richardson DM (2023) Disentangling the relationships among abundance, invasiveness and invasibility in trait space. NPJ Biodivers 2:13. 10.1038/s44185-023-00019-1 39242656 PMC11332024

[28] Ishtiaq F, Swain S, Kumar SS (2021) *Anopheles stephensi* (Asian Malaria Mosquito). Trends Parasitol 37:571–572. 10.1016/j.pt.2021.03.009 33865712

[29] Juliano SA (2009) Species interactions among larval mosquitoes: context dependence across habitat gradients. Annu Rev Entomol 54:37–56. 10.1146/annurev.ento.54.110807.090611 19067629 PMC2664081

[30] Kahamba NF, Limwagu AJ, Mapua SA, Msugupakulya BJ, Msaky DS, Kaindoa EW, Ngowo HS, Okumu FO (2020) Habitat characteristics and insecticide susceptibility of *Aedes aegypti* in the Ifakara area, south-eastern Tanzania. Parasit Vectors 13:53. 10.1186/s13071-020-3920-y 32033619 PMC7006121

[31] Li Y, Zhou G, Zhong D, Wang X, Hemming-Schroeder E, David RE, Lee MC, Zhong S, Yi G, Liu Z, Cui G, Yan G (2021) Widespread multiple insecticide resistance in the major dengue vector *Aedes albopictus* in Hainan Province, China. Pest Manag Sci 77:1945–1953. 10.1002/ps.6222 33301644 PMC7986907

[32] Marini G, Guzzetta G, Baldacchino F, Arnoldi D, Montarsi F, Capelli G, Rizzoli A, Merler S, Rosà R (2017) The effect of interspecific competition on the temporal dynamics of *Aedes albopictus* and *Culex pipiens*. Parasit Vectors 10:102. 10.1186/s13071-017-2041-8 28228159 PMC5322594

[33] Merga H, Degefa T, Birhanu Z, Abiy E, Lee MC, Yan G, Yewhalaw D (2024) Urban malaria and its determinants in Eastern Ethiopia: the role of *Anopheles stephensi* and urbanization. Malar J 23:303. 10.1186/s12936-024-05126-3 39385192 PMC11465532

[34] Musunzaji PS, Ndenga BA, Mzee S, Abubakar LU, Kitron UD, Labeaud AD, Mutuku FM (2023) Oviposition preferences of *Aedes aegypti* in Msambweni, Kwale County, Kenya. J Am Mosq Control Assoc 39:85–95. 10.2987/22-7103 37270926 PMC10885850

[35] Mwingira VS, Mboera LEG, Takken W (2021) Synergism between nonane and emanations from soil as cues in oviposition-site selection of natural populations of *Anopheles gambiae* and *Culex quinquefasciatus*. Malar J 20:52. 10.1186/s12936-020-03575-0 33478526 PMC7819190

[36] Noden BH, O'Neal PA, Fader JE, Juliano SA (2016) Impact of inter- and intra-specific competition among larvae on larval, adult, and life-table traits of *Aedes aegypti* and *Aedes albopictus* females. Ecol Entomol 41:192–200. 10.1111/een.12290 27141149 PMC4850917

[37] Parker C, Ramirez D, Connelly CR (2020) State-wide survey of *Aedes aegypti* and *Aedes albopictus* (Diptera: Culicidae) in Florida. J Vector Ecol 44: 210–215. 10.1111/jvec.12351 31729793

[38] President’s Malaria Initiatives. (2020) PMI VectorLink Ethiopia Project Final Entomology Report May 2019-March 2020. Rockville, MD: Abt Associates.

[39] President’s Malaria Initiatives. (2023) PMI VectorLink Ethiopia Final Entomological Report, April 2022-March 2023. Rockville, MD: Abt Associates.

[40] Reiskind MH, Zarrabi AA, Lounibos LP (2012) Effects of combination of leaf resources on competition in container mosquito larvae. Bull Entomol Res 102:424–434. 10.1017/S0007485311000861 22314102 PMC3401315

[41] Seid M, Aklilu E, Animut A (2024) Spatio-temporal occurrence and habitat characteristics of Aedes aegypti (Diptera: Culicidae) larvae in Southern Afar region, Ethiopia. Trop Med Health 52:51. 10.1186/s41182-024-00612-5 39095931 PMC11295501

[42] Sinclair D, Gogtay N, Brand F, Olliaro P (2011) Artemisinin-based combination therapy for treating uncomplicated *Plasmodium vivax* malaria. Cochrane Database Syst Rev 6:CD008492. 10.1002/14651858.CD008492.pub2 21735431

[43] Sinka ME, Bangs MJ, Manguin S, Chareonviriyaphap T, Patil AP, Temperley WH, Gething PW, Elyazar IR, Kabaria CW, Harbach RE, Hay SI (2011) The dominant *Anopheles* vectors of human malaria in the Asia-Pacific region: occurrence data, distribution maps and bionomic précis. Parasit Vectors 4:89. 10.1186/1756-3305-4-89 21612587 PMC3127851

[44] Sinka ME, Pironon S, Massey NC, Longbottom J, Hemingway J, Moyes CL, Willis KJ (2020) A new malaria vector in Africa: Predicting the expansion range of *Anopheles stephensi* and identifying the urban populations at risk. Proc Natl Acad Sci U S A 117:24900–24908. 10.1073/pnas.2003976117 32929020 PMC7547157

[45] Takken W, Charlwood D, Lindsay SW (2024) The behaviour of adult *Anopheles gambiae*, sub-Saharan Africa's principal malaria vector, and its relevance to malaria control: a review. Malar J 23:161. 10.1186/s12936-024-04982-3 38783348 PMC11112813

[46] Talaga S, Dejean A, Mouza C, Dumont Y, Leroy C (2018) Larval interference competition between the native Neotropical mosquito *Limatus durhamii* and the invasive *Aedes aegypti* improves the fitness of both species. Insect Sci 25:1102–1107. 10.1111/1744-7917.12480 28497885

[47] Tenywa FSC, Musa JJ, Musiba RM, Swai JK, Mpelepele AB, Okumu FO, Maia MF (2022) Evaluation of an ivermectin-based attractive targeted sugar bait (ATSB) against *Aedes aegypti* in Tanzania. Wellcome Open Res 7:4. 10.12688/wellcomeopenres.17442.1 37409221 PMC10318376

[48] Thomas S, Ravishankaran S, Justin JA, Asokan A, Mathai MT, Valecha N, Thomas MB, Eapen A (2016) Overhead tank is the potential breeding habitat of *Anopheles stephensi* in an urban transmission setting of Chennai, India. Malar J 15:274. 10.1186/s12936-016-1321-7 27169513 PMC4865005

[49] Vatandoost H, Oshaghi MA, Abaie MR, Shahi M, Yaaghoobi F, Baghaii M, Hanafi-Bojd AA, Zamani G, Townson H (2006) Bionomics of *Anopheles stephensi* Liston in the malarious area of Hormozgan province, southern Iran, 2002. Acta Trop 97:196–203. 10.1016/j.actatropica.2005.11.002 16329986

[50] World Health Organization (2022) WHO initiative to stop the spread of *Anopheles stephensi* in Africa. Geneva: WHO.

[51] Yan J, Mackay AJ and Stone CM (2024) Dynamics of invasive mosquitoes: introduction pathways, limiting factors, and their potential role in vector-borne pathogen transmission. Front Trop Dis 5:1503120. 10.3389/fitd.2024.1503120

[52] Yared S, Gebressilasie A, Worku A, Mohammed A, Gunarathna I, Rajamanickam D, Waymire E, Balkew M, Carter TE (2024) Breeding habitats, bionomics and phylogenetic analysis of *Aedes aegypti* and first detection of *Culiseta longiareolata*, and *Ae. hirsutus* in Somali Region, eastern Ethiopia. PLoS One 19:e0296406. 10.1371/journal.pone.0296406 38165914 PMC10760653

[53] Zhao M, Ran X, Bai Y, Ma Z, Gao J, Xing D, Li C, Guo X, Jian X, Liu W, Liao Y, Chen K, Zhang H, Zhao T (2023) Genetic diversity of *Aedes aegypti* and *Aedes albopictus* from cohabiting fields in Hainan Island and the Leizhou Peninsula, China. Parasit Vectors. 16:319. 10.1186/s13071-023-05936-5 37684698 PMC10486073

[54] Zhou G, Zhong D, Yewhalaw D, Yan G (2024) *Anopheles stephensi* ecology and control in Africa. Trends Parasitol 40:102–105. 10.1016/j.pt.2023.11.011 38142196 PMC11849806

